# 慢性阻塞性肺疾病合并原发性支气管肺癌118例临床特征分析

**DOI:** 10.3779/j.issn.1009-3419.2017.08.06

**Published:** 2017-08-20

**Authors:** 松林 赵, 秀红 聂, 霖 张, 威 张, 汉 肖

**Affiliations:** 100053 北京，首都医科大学宣武医院呼吸科 Department of Respiratory Medicine, Xuanwu Hospital, Capital Medical University, Beijing 100053, China

**Keywords:** 慢性阻塞性肺疾病, 原发性支气管肺癌, 临床特征, Chronic obstructive pulmonary disease, Primary bronchopulmonary carcinoma, Clinical characteristics

## Abstract

**背景与目的:**

探讨慢性阻塞性肺疾病（chronic obstructive pulmonary disease, COPD）合并原发性支气管肺癌的临床特征，以便临床早期诊断COPD合并原发性支气管肺癌患者。

**方法:**

回顾性分析我院2013年1月-2016年12月118例COPD合并原发性支气管肺癌患者的临床资料，包括年龄、性别、吸烟史、吸烟指数、临床症状及体征、病理类型、分期、转移部位及肺功能指标，选择同时期的120例单纯性COPD患者作为对照。

**结果:**

COPD合并肺癌组患者吸烟率（55.1%）及吸烟指数≥400支/年患者比例（90.8%）均高于单纯COPD组患者（20.8%, 48.0%），组间差异有统计学意义（*P* < 0.01）；COPD合并肺癌组患者常见症状如咳嗽、咳痰、发热、乏力、呼吸困难发生率与单纯COPD组患者相比无统计学差异（*P* > 0.05），而咯血、消瘦、胸痛、声音嘶哑、胸腔积液、肺不张发生率均显著高于单纯COPD组患者，差异有统计学意义（*P* < 0.01）；COPD合并肺癌组患者首次诊断时63.6%为晚期或局部晚期，远处转移以胸膜转移和骨转移多见；两组患者之间第一秒用力呼气容积（forced expiratory volume in one second, FEV_1_）与用力肺活量（forced vital capacity, FVC）比值（FEV_1_/FVC）、FEV_1_占预计值的百分比（FEV_1_% pre）、肺总量（total lung capacity, TLC）、残气容积（residual volume, RV）与TLC的比值（RV/TLC）无明显差异（*P* > 0.05），但COPD合并肺癌组患者的一氧化碳弥散量（diffusing capacity of carbon monoxide, DLCO）较单纯COPD组患者降低，差异有统计学意义（*P* < 0.05）；COPD合并肺癌组患者病理类型以鳞癌最多见（51.7%），其中男性患者以鳞癌（60.7%）为主，而女性患者以腺癌（69.0%）为主。

**结论:**

COPD合并原发性支气管肺癌好发于男性吸烟患者，鳞癌发生率高，早期临床表现缺乏特异性，首次诊断时多为晚期或局部晚期，定期对COPD患者行胸部CT检查可以尽早发现肺癌。

## Abstract (en)

**Background and objective:**

The aim of this study is to investigate the clinical characteristics of patients with primary bronchopulmonary carcinoma complicated with chronic obstructive pulmonary disease (COPD), and to optimize the early diagnoses in the coexistence of COPD and primary bronchopulmonary carcinoma.

**Methods:**

The clinical data of 118 patients with COPD complicated with primary bronchopulmonary carcinoma were analyzed retrospectively, including age, sex, smoking history, smoking index, clinical symptoms and signs, pathological type, staging, metastasis site and lung function index. 120 patients with simple COPD were selected as control.

**Results:**

The smoking rate (55.1%) and smoking index ≥400 branch /year (90.8%) of the patients with COPD complicated with primary bronchopulmonary carcinoma were higher than the simple COPD group (20.8%, 48.0%). The difference between the two groups was statistically significant (*P* < 0.01). There were no significant differences in the incidence of common symptoms such as cough, sputum, fever, fatigue and dyspnea in COPD complicated with primary bronchopulmonary carcinoma patients with simple COPD group (*P* > 0.05), while the incidence of hemoptysis, weight loss, chest pain, hoarseness, pleural effusion and atelectasis were significantly higher than those in simple COPD group (*P* < 0.01). When the patients were first diagnosed as COPD with primary bronchopulmonary carcinoma, 63.6% of the group were advanced or located late, and the distant metastases are common for pleural metastasis and bone metastases. There was no significant difference in forced expiratory volume in one second/forced vital capacity (FEV_1_/FVC), FEV_1_% pre, total lung capacity (TLC) and residual volume (RV)/TLC between the two groups (*P* > 0.05), but the diffusing capacity of carbon monoxide (DLCO) of COPD patients complicated with primary bronchopulmonary carcinoma was lower than that of simple COPD patients (*P* < 0.05). In the COPD patients with primary bronchopulmonary carcinoma, squamous cell carcinoma was the most common pathological type (51.7%). Male patients were mainly squamous cell carcinoma (60.7%), while female patients with adenocarcinoma (69.0%).

**Conclusion:**

COPD combined with primary bronchopulmonary carcinoma occurs in male smokers more. There is higher incidence of squamous cell carcinoma. When they are first diagnosed, most of them are advanced or located late, due to no specific clinical symptoms at the early stages. Periodic chest CT examination for COPD patients can help early diagnoses of primary bronchopulmonary carcinoma.

慢性阻塞性肺疾病（chronic obstructive pulmonary disease, COPD）是一种以不完全可逆气流受限为主要特征的可防可治的慢性呼吸系统疾病。原发性支气管肺癌也是呼吸系统的一种常见病与多发病，发病率和死亡率均居我国恶性肿瘤的首位^[1]^。Young等^[2]^研究认为，吸烟、遗传、环境、免疫反应异常等一些共同的危险因素可以影响COPD和肺癌的发生发展，而且COPD是肺癌的独立危险因素^[3]^。COPD和肺癌早期的临床表现极为相似，缺乏特异性，容易造成误诊、漏诊。本研究对2013年1月-2016年12月首都医科大学宣武医院收治的118例COPD合并肺癌患者的临床资料进行回顾性分析，比较COPD合并肺癌患者与单纯性COPD患者临床特征的异同，以指导临床早期诊断COPD合并肺癌患者。

## 1 资料与方法

### 1.1 一般资料

选取2013年1月-2016年12月在我院住院治疗的118例COPD合并肺癌患者，入选患者均临床资料完整，并选择同期在我院治疗的120例单纯性COPD患者的临床资料进行对比研究。慢阻肺的诊断符合中华医学会呼吸疾病学会制定的《慢性阻塞性肺疾病诊治规范》^[4]^中的诊断标准，肺功能使用美国森迪斯Vmax229肺功能仪测定。所有肺癌患者均经病理组织学确诊，按国际肺癌协会2009年第七版分期标准进行肿瘤-淋巴结-转移（tumor-node-metastasis, TNM）分期^[5]^。COPD合并肺癌患者年龄42岁-91岁，平均（72.38±11.26）岁，男性89例，女性29例；单纯性COPD患者年龄44岁-87岁，平均（69.35±10.85）岁，男性85例，女性35例。两组患者年龄、性别比较，差异无统计学意义（*P* > 0.05）。纳入研究对象意识清楚，无精神障碍，除外患有严重心肝肾脏疾病患者。

### 1.2 方法

采用回顾性分析的方法对患者的病例资料进行统计分析，统计内容包括年龄、性别、吸烟史、吸烟指数、临床症状及体征（咳嗽、咳痰、咯血、发热、胸痛、呼吸困难、乏力、消瘦、声音嘶哑、胸腔积液、肺不张等）、病理类型、分期、转移部位，肺功能指标主要包括：第一秒用力呼气容积（forced expiratory volume in one second, FEV_1_）与用力肺活量（forced vital capacity, FVC）比值（FEV_1_/FVC）、FEV_1_占预计值的百分比（FEV_1_% pre）、肺总量（total lung capacity, TLC）、残气容积（residual volume, RV）与TLC的比值（RV/TLC）、一氧化碳弥散量（diffusing capacity of carbon monoxide, DLCO）。

### 1.3 统计学方法

采用SPSS 18.0软件进行统计分析，计量资料以均数±标准差（Mean±SD）表示，采用*t*检验，计数资料采取卡方检验，*P* < 0.05为差异有统计学意义。

## 2 结果

### 2.1 COPD合并肺癌组患者与单纯COPD组患者吸烟情况的比较

COPD合并肺癌组患者的吸烟率及吸烟指数均显著高于单纯COPD组患者，差异有统计学意义（*P* < 0.01）（表 1）。

**1:** 两组患者吸烟情况的比较[*n* (%)] Comparison of smoking index between the two groups of patients [*n* (%)]

Group	Number of smokers			Smoking index (branch/year)	
	Yes	No		≥400	< 400	
COPD with bronchopulmonary carcinoma (*n*=118)	65(55.1)	53 (44.9)		59 (90.8)	6 (9.2)
Simple COPD (*n*=120)	25(20.8)	95 (79.2)		12 (48.0)	13 (52.0)
*X*^2^	27.34			16.64	
*P*	< 0.01			< 0.01	
Smoking is less than 3 years for smoking cessation or smoking ≥1/day and for ≥1 year. COPD: chronic obstructive pulmonary disease.					

### 2.2 COPD合并肺癌组患者与单纯COPD组患者临床症状及体征的比较

COPD合并肺癌组患者咯血、消瘦、胸痛、声音嘶哑、胸腔积液、肺不张等临床症状或体征发生率均显著高于单纯COPD组患者，差异均有统计学意义（*P* < 0.01），而咳嗽、咳痰、发热、乏力、呼吸困难等症状发生率组间无差异（*P* > 0.05）（表 2）。

**2:** COPD合并肺癌组患者与单纯COPD组患者临床症状及体征发生率的比较[*n* (%)] Clinical symptoms and signs of the rate of comparison between COPD patients with primary bronchopulmonary carcinoma and simple COPD patients [*n* (%)]

Clinical symptoms and signs	COPD with bronchopulmonary carcinoma (*n*=118)	Simple COPD (*n*=120)	*X*^2^	*P*
Cough	109 (92.4)	112 (93.3)	0.13	0.71
Sputum	101 (85.6)	105 (87.5)	0.07	0.78
Hemoptysis	71 (60.2)	10 (8.3)	73.51	< 0.01
Fever	33 (28.0)	26(21.7)	1.17	0.28
Fatigue	23 (19.5)	12 (10.0)	3.63	0.06
Dyspnea	47 (39.8)	29 (24.2)	2.86	0.09
Weight loss	48 (40.7)	5 (4.2)	49.99	< 0.01
Chest pain	70 (59.3)	20(16.7)	43.68	< 0.01
Hoarseness	6(5.1)	0 (0.0)	9.97	< 0.01
Pleural effusion	42 (35.6)	12 (10.0)	22.38	< 0.01
Atelectasis	36 (30.5)	3 (2.5)	39.28	< 0.01

### 2.3 COPD合并肺癌组患者分期与转移部位

根据国际肺癌协会2009年第七版分期标准进行TNM分期：Ⅰ期3例（2.5%），Ⅱ期13例（11.0%），Ⅲa期27例（22.9%），Ⅲb期38例（32.2%），Ⅳ期37例（31.4%），晚期及局部晚期（Ⅲb期）比例高达63.6%。首次确诊肺癌时远处转移部位有67处，胸膜转移最多（32例），其次是骨转移（19例）、肝转移（8例）、脑转移（6例）及肾上腺转移（2例）。

### 2.4 COPD合并肺癌组患者与单纯COPD组患者肺功能主要指标的比较

两组患者之间FEV_1_/FVC、FEV_1_% pre、TLC、RV/TLC无明显差异（*P* > 0.05），但COPD合并肺癌组患者的DLCO较单纯COPD组患者降低，差异有统计学意义（*P* < 0.05）（表 3）。

**3:** COPD合并肺癌组患者与单纯COPD组患者肺功能主要指标的比较（Mean±SD, %） Comparison of the main indicators of lung function between COPD patients with primary bronchopulmonary carcinoma and simple COPD patients (Mean±SD, %)

Lung function index	COPD with bronchopulmonary carcinoma (*n*=118)	Simple COPD (*n*=120)	*t*	*P*
FEV_1_/FVC	48.76±11.03	50.18±12.42	1.54	0.11
FEV_1_% pre	45.18±15.44	48.31±17.23	1.18	0.12
TLC	79.36±9.72	82.76±11.75	2.97	0.08
RV/TLC	48.38±9.42	51.36±8.72	3.46	0.07
DLCO	63.38±10.78	70.48±10.57	6.75	< 0.05
FEV_1_: forced expiratory volume in one second; FVC: forced vital capacity; TLC: total lung capacity; RV: residual volume; DLCO: diffusing capacity of carbon monoxide.				

### 2.5 COPD合并肺癌组患者的病理类型

COPD合并肺癌组患者病理类型以鳞癌最多见（51.7%），其中男性患者以鳞癌（60.7%）为主，而女性患者以腺癌（69.0%）为主（*P* < 0.01）（表 4）。

**4:** COPD合并肺癌组患者不同性别病理类型的比较[*n*(%)] Comparison of COPD patients with bronchopulmonary carcinoma in differences in sex and pathological types [*n*(%)]

Pathological types	Total (*n*=118)	Male (*n*=89)	Female (*n*=29)	*X*^2^	*P*
Squamous cell carcinoma	61 (51.7)	54 (60.7)	7 (24.1)	35.75	< 0.01
Adenocarcinoma	35 (29.7)	15(16.9)	20 (69.0)		
Other non small cell carcinoma	2 (1.7)	2 (2.2)	0 (0.0)		
Small cell carcinoma	20(16.9)	18 (20.2)	2 (6.9)		

## 3 讨论

慢性阻塞性肺疾病和肺癌都是呼吸系统的高发性疾病，死亡率也高。研究^[6, 7]^认为，吸烟、遗传、环境、免疫反应异常等一些共同的危险因素可以影响COPD和肺癌的发生与发展，其中吸烟的证据最明确，吸烟是目前公认的COPD与肺癌的重要的共同致病因素。烟草中含有苯并芘、尼古丁、焦油、亚硝胺等多种氧化剂和致癌物，这些物质不仅可以引起COPD的发生与发展，烟草中的致癌物也可以引起支气管黏膜上皮细胞腺瘤样增生、鳞状上皮化生、DNA损伤而导致癌变^[8, 9]^。研究^[10]^表明，吸烟可以使慢阻肺患者发生肺癌的风险明显增加，达到肺功能正常的吸烟者的4倍-6倍。吸烟指数与肺癌发病率密切相关，吸烟指数越高，患肺癌的几率越大。有研究^[11]^报道，吸烟量≥400支/年的COPD患者肺癌的发病率明显高于吸烟量 < 400支/年的COPD患者，本研究结果显示COPD合并肺癌组患者的吸烟率及吸烟指数均显著高于单纯COPD组患者，提示吸烟可以导致COPD患者发生肺癌的几率增加。

本研究结果显示咳嗽、咳痰、发热、乏力、呼吸困难等症状可以是COPD合并肺癌及单纯性COPD患者共有的症状，缺乏特异性，而COPD合并肺癌患者咯血、消瘦、胸痛、声音嘶哑、胸腔积液、肺不张等临床症状或体征均显著高于单纯COPD患者，表明如果COPD患者出现上述症状或体征时，应高度警惕癌变的可能，本研究还发现COPD合并肺癌患者首次诊断时晚期及局部晚期已达63.6%。肺癌早期诊断尤为重要，研究^[12]^报道低剂量胸部CT筛查是肺癌早期诊断的重要方法，可使肺癌病死率减低20%，定期对COPD患者行胸部CT检查可以尽早发现肺癌，避免出现漏诊、误诊。

肺癌可以压迫病变部位的支气管引起阻塞，导致阻塞性通气功能障碍。韩勇等^[13]^研究发现，肺癌组FEV_1_与正常对照组相比明显下降，提示肺功能的改变（FEV_1_下降）是肺癌的独立的危险因素。此外，也有研究显示，肺气肿可以导致发生肺癌的风险增加3倍-6倍，并且与预后相关，肺气肿病变程度越重，肺癌患者病死率越高^[14]^。陈亚红^[15]^的研究发现，肺癌患者明显的肺功能改变要经过近十年的潜伏期才能出现，而肺癌的自然病程远低于此，所以COPD合并肺癌患者出现明显肺功能改变的可能性很小。本研究结果显示，COPD合并肺癌组患者与单纯COPD组患者FEV_1_/FVC、FEV_1_%pre以及TLC、RV/TLC等指标并无明显差异，进一步证实此观点。同时，本研究结果显示COPD合并肺癌组患者的DLCO较单纯COPD组患者明显减低，与魏浩成等^[16]^文献报道相似，说明肺癌对患者的弥散功能有明显的影响，究其原因可能与肺癌病理改变引起通气/血流比例失调进一步加重有关。

肺癌病理类型分为小细胞癌和非小细胞癌，后者主要包括鳞癌、腺癌、大细胞癌和腺鳞癌等。本研究发现COPD合并肺癌患者以鳞癌最多见（51.7%），尤其在男性中比例高达60.7%，而近来世界卫生组织和我国的学者发现近30年来腺癌有显著增加趋势位居第一，鳞癌约占肺癌总数的30%左右^[17]^，可能与本研究中男性患者居多且吸烟率高有关。本研究中COPD合并肺癌患者中腺癌占29.7%，其中女性患者腺癌比率为69%，究其原因可能与女性患者接触生物燃料而吸烟率低有关。

总之，吸烟是慢阻肺和肺癌共同的发病危险因素，吸烟指数与肺癌发病率密切相关，戒烟可以降低慢阻肺患者肺癌的发生率。当慢阻肺患者出现咯血、消瘦、胸痛、声音嘶哑等临床症状和（或）胸腔积液、肺不张等影像改变时，应及时行痰脱落细胞学、纤维支气管镜等检查，提高肺癌的诊断意识。
